# Can Positive Social Contact Encourage Residents’ Community Citizenship Behavior? The Role of Personal Benefit, Sympathetic Understanding, and Place Identity

**DOI:** 10.3390/bs14040307

**Published:** 2024-04-10

**Authors:** Yaxi Wang, Bo Wu, Jiaqi Li, Qing Yuan, Nan Chen

**Affiliations:** 1 Research Institute for Study Travel, Henan University, Kaifeng 475001, China; wangyaxii@126.com (Y.W.); 20211007@zzut.edu.cn (J.L.); yuanqing@henu.edu.cn (Q.Y.); 2 Department of Leisure Service & Sports, Pai Chai University, Daejeon 35345, Republic of Korea; 3 School of Culture and Tourism, Henan University, Kaifeng 475001, China; woopoo@henu.edu.cn; 4 School of Management, Zhengzhou University of Technology, Zhengzhou 450044, China

**Keywords:** community citizenship behavior, the egoistic–altruistic motivation framework, positive social contact, personal benefit, sympathetic understanding, place identity

## Abstract

Identified as an increasingly pivotal aspect, the benevolent extra-role characteristic of community citizenship behavior contributes to destination development efficiency and social cohesion. Based on the egoistic–altruistic motivation framework, this study investigated three motivations that propel residents to exercise community citizenship behaviors in a positive social contact context, namely self-focused, other-focused, and place-focused motivation. A conceptual model combined with positive contact, personal benefit, sympathetic understanding, place identity, and community citizenship behavior was developed and tested using partial least squares structural equation modeling (PLS-SEM) through data from 366 residents in Kaifeng, China. The findings showed that of the three motivations for community citizenship behaviors, place identity contributed the most, and personal benefits failed to predict community citizenship behaviors. Furthermore, sympathetic understanding with tourists was most fostered by residents from the perception of positive contact with tourists. These findings offer a novel theoretical framework for scholarly investigation and provide practical insights for tourism managers regarding strategies to influence residents’ community citizenship behavior.

## 1. Introduction

Considering the essential role of residents in evaluating tourism sustainability, supporting effective tourism planning, influencing tourists’ experiences, and serving as a promotional signboard, it is evident that residents play a crucial role in local tourism development [1,2,3]. In this respect, cultural tourism, centered around history and culture, necessitates significant content processing and expression to offer tourists the anticipated cultural experience, involving substantial human and material resources, with tourism performing arts as common examples. Beyond the tourist activity areas, symbols of local beliefs, behaviors, and norms observed through the tourists’ gaze are vital for projecting authentic local culture and satisfying tourists’ cultural expectations [4]. However, there is a notable lack of research richness in understanding destination residents’ perceptions of interaction and their diverse behaviors compared to their critical functions in this context.

Fortunately, academics have expressed interest in the novel concept of community citizenship behavior (CCB) lately [5,6]. This concept aligns with the evolving research trend where scholars are shifting focus from supporting destination residents to investigating a broader spectrum of resident behaviors [7,8]. Additionally, the altruistic extra-role characteristic of CCB can significantly enhance destination development efficiency and social cohesion, making it a burgeoning aspect. According to Xie, Zhao, and Ma [9], active participation in tourism development allows residents to cultivate a sense of being a host, local identity, pride, and immediate subjective well-being. These positive outcomes heighten the potential for establishing a mutually beneficial relationship between residents and local tourism development. Moreover, these favorable effects will also greatly improve the tourist’s experience and satisfaction, giving a big boost to subsequent revisits and willingness to recommend [2,3]. Consequently, addressing how to motivate residents to engage in community citizenship behavior becomes a crucial and relevant topic.

Regarding the precursors of community citizenship behavior, this study adopted a motivational perspective. In contrast to the prevalent focus on psychological perception or attitude in related studies, motivation involves more potent energy and precise direction, elucidating significant variations in prosocial behaviors influenced by individual differences in personality traits and effects [10]. Specifically, the egoistic–altruistic motivation framework, where egoistic (self-oriented) motivation involves pursuing personal benefit and altruistic (other-oriented) motivation reflects concern for others’ welfare, has frequently been employed to explore the triggers of pro-tourism behaviors among tourists or residents [11,12,13,14]. Results from these studies remain contradictory and inconclusive [15,16,17,18], which may be attributed to the lack of clarity about the context in which motivation arises [19]. According to Ling and Xu [20], while motivation is an internal psychological need stemming from internal desires and interests, it is also easily influenced by external situations. In other words, the direction and level for a person’s motivation for a specific behavior may fluctuate based on changes in their circumstances. Recognizing how scenarios can impact motivation is crucial as it enables individuals to identify and address factors that can either boost or impede their motivation levels, an aspect that has received limited attention thus far. 

To accurately investigate residents’ motivations for participating in community citizenship behavior, this study concentrated on positive contact as perceived by residents. Given that residents and tourists are the two largest groups in a tourist destination, interaction between them is inevitable. Building a good rapport between locals and tourists is a never-ending goal for managers and a prerequisite for successful tourism. Much like how tourists evaluate their experience based on the hospitality of residents, residents decide whether to assist and support tourists depending on the latter’s respect for the local culture and way of life [21,22]. A positive contact can be beneficial not only to tourists but also residents. Specifically, favorable assessments of the interaction with tourists can enhance residents’ perception of tourists and provide residents with chances to reflect on their homeland, leading to a strong sense of pride and well-being. However, in contrast to the extensive research on resident–tourist interaction from the tourist perspective, as noted by Tse and Tung [23], there is a notable lack of breadth and depth in research on the resident’s viewpoint.

By taking into consideration of the above research gaps, this study aims to advance research by exploring the antecedents of CCB within a positive contact scenario grounded in the egoistic–altruistic motivation framework. In particular, this study marks the first attempt to incorporate the three variables—perceived benefits, sympathetic understanding, and place identity—into the ego-altruism theoretical framework, where they are categorized as self-interested, other-interested, and place-interested motives, respectively. Accordingly, the objectives of this study are as follows:

(1) investigate the potential of positive contact in encouraging residents’ community citizenship behavior;
(2) assess the influence of the three aforementioned motivations on promoting community citizenship behavior;
(3) examine the mediating effect of these motivations on the relationship between positive contact and residents’ community citizenship behavior.

Theoretically, the empirical findings significantly contribute to understanding community citizenship behavior from a motivational standpoint and enhance knowledge of altruistic motivation by categorizing it into two types—other-focused and place-focused—aligning more closely with the characteristics of a tourist destination. Furthermore, our study underscores the crucial role of residents in resident–tourist interaction, offering valuable insights that complement research on resident perception and behavior. The managerial implications highlight the potential role of residents in participating in tourism development and maintaining positive resident–tourist relationships.

## 2. Literature Review and Hypotheses Development

### 2.1. Community Citizenship Behavior

Citizenship behavior holds crucial significance in the realms of consumer behavior and organizational management due to the spontaneous altruistic actions of stakeholders, contributing to the operational efficiency of the organization [11]. The concepts of customer and organizational citizenship behavior have been extensively researched in recent decades [24,25,26,27]. Scholars have drawn parallels between customers and employees in business organizations and tourists and residents in a tourist destination, emphasizing corresponding characteristics [5]. Accordingly, community citizenship behavior, derived from the organizational citizenship behavior concept and considering the destination as a unified organization, has become a research hotspot. According to Zhang and Xu [5], and Wu et al. [6], community citizenship behavior encompasses discretionary and positive behaviors by residents contributing to the destination’s success and sustainability without direct rewards. 

Despite the similarity of the definitions, the statements (e.g., resident citizenship behavior [28], place citizenship behavior [5], community citizenship behavior [6], and pro-tourism citizenship behavior [29]) and dimensions of community citizenship behavior exhibit inconsistencies. In the present study, we combined the findings from Zhang and Xu [5], and Wu et al. [6], to categorize community citizenship behavior into involving behavior, recommending behavior, tourist-helping behavior, resident-helping behavior, and protecting behavior. Resident-helping and protecting behaviors are associated with efforts in tourism development, encompassing activities such as reporting issues related to local tourism development, maintaining neighborly relationships, and safeguarding tourism resources. Recommending and tourist-helping behaviors are directed towards tourists or potential tourists, such as suggesting tourist attractions and products and assisting tourists.

While a limited number of studies have investigated the determinants of community citizenship behavior or its sub-dimensions, they predominantly build on the foundation of resident support research. Influencing factors include perceptions and evaluations of the place, such as perceived tourism impacts [18], perceived justice [30], place attachment [31], destination psychological ownership [5], etc., and the evaluation of the personal initiative, including self-efficacy [32], emotion [33], values [20], etc. These studies align with established theoretical frameworks like social exchange theory, place attachment theory, and emotional solidarity theory, with a scant focus on the motivational lens [34]. Indeed, uncovering the procedural mechanisms of resident behavior from a motivational standpoint would furnish management with more precise and actionable recommendations, an aspect where there is a slight deficiency in related research. Moreover, as outlined by Xu, Xue, and Gursoy [35], the compassion residents acquire through interactions with the destination, tourists, or other residents can notably forecast community citizenship behavior. Few studies have hitherto focused on the importance of host–guest interaction concerning residents and its impact on predicting community citizenship behavior [35].

### 2.2. Resident–Tourist Social Contact

The notion of social contact originates from Allport’s (1954) contact theory, focusing on the role of intergroup contact in diminishing prejudice between culturally diverse groups [36]. Within the context of tourism research, resident–tourist social contact, typically measured in terms of quantity and quality, represents a pivotal area of investigation [37,38]. Scholars have demonstrated that positive social contact yields favorable outcomes for both tourists and residents [21]. For instance, pleasant interactions can diminish tourists’ perceived cultural distance [21], and residents can gain a renewed perspective on the impact of tourism development [37]. These positive responses contribute to fostering a harmonious relationship between the two groups, ultimately benefiting tourism development.

While the majority of research on the link between resident–tourist contact and positive outcomes has been conducted from the tourists’ perspective, understanding intergroup relations from the resident viewpoint is crucial. It elucidates how residents perceive tourists and how this perception influences their subsequent behaviors [2,22,35]. Residents do not solely rely on evaluations of tourism development impacts to decide on pro-tourism behaviors, such as recommending the destination or assisting tourists. A period of positive social contact is adequate for residents to respond positively based on their current judgments about interpersonal relationships [21,35,39]. For example, Wang, Xiong, and Gage [39] validated the causal relationship between residents’ destination brand-supporting behaviors and their perception of the interaction quality with tourists, emphasizing that a positive interaction quality perception encourages residents to act as destination ambassadors. Li et al. [40] found that in cross-cultural encounters, residents’ cultural intelligence significantly stimulates their helping and tolerant behaviors toward tourists. Since advocacy behavior, helping behavior, and tolerant behavior all fall under the umbrella of community citizenship behavior, it is logical to propose the following hypotheses:

**H1a.**  *Resident–tourist interaction behavior can positively influence community citizenship behavior.*

**H1b.**  *Resident–tourist interaction quality can positively influence community citizenship behavior.*

### 2.3. The Egoistic–Altruistic Motivation Framework of CCB

Rioux and Penner [24] were the first to introduce a motivational approach to investigate the determinants of organizational citizenship behavior (OCB), asserting that three crucial motivational factors—prosocial values, organizational concerns, and impression management—underlie OCB. Subsequent research has widely supported this motivational approach [11]. Expanding on earlier works, some scholars have applied an other-orientation (versus self-orientation) theory using a multifocal lens, attempting to categorize motivations for citizenship behavior within the egoistic–altruistic motivation framework [11,41,42]. This framework has also found extensive application in understanding motivations for volunteering, photo sharing, and charitable donating behaviors. For instance, Butts et al. [43] explored the compassion fade phenomenon, identifying empathetic concern, perceived impact, and anticipated positive affect as other-oriented and self-oriented motivations in triggering helping behavior. The egoistic–altruistic motivation framework has spurred research into the motivations behind community citizenship behavior, given its similarities with other discretionary prosocial behaviors, offering fresh perspectives in community citizenship behavior research. 

In tourism research, studies on behavioral motivation are prevalent, particularly for altruistic behaviors like pro-environmental behavior, helping behavior, volunteering behavior, and destination-responsible behavior [16]. In addition to the extrinsic versus intrinsic motivation framework, the self-oriented versus other-oriented motivation framework plays a significant role in these studies. Scholars often consider the primary driver of altruistic behavior in tourism to be perceived benefits or values driven by selfish instincts. However, this dominant focus on self-interest-oriented motivation overlooks people’s social instincts and their willingness to prioritize broader social or group interests [15]. On the contrary, Paraskevaidis and Andriotis introduced the altruistic surplus phenomenon into tourism to balance the social exchange theory’s excessive emphasis on individual interests [16]. In recent years, several studies have explored other-oriented motives such as place identity [44,45], altruism [19,46], and empathy [33,47], collectively elucidating the behavior of tourists and residents in tourist destinations. Building on this foundation, this study selects three motives under the egoistic–altruistic motivation framework: personal benefits from tourism development, sympathetic understanding, and place identity as the egoistic-oriented, other-oriented, and place-oriented motives, respectively. 

#### 2.3.1. Personal Benefits from Tourism Development

Personal benefits from tourism development represent a classic self-interest-oriented motivational concept [48], given that when residents perceive personal benefits to outweigh costs, they tend to positively assess the impacts of tourism development and engage in actions to maintain the current level of access, such as supporting tourism. Conversely, a negative assessment leads to refusal or avoidance behaviors. Past studies typically categorize personal benefits into personal economic, sociocultural, and environmental benefits based on content [1,49] and economic and non-economic benefits based on value attributes [50].

Positive social contact naturally prompts residents to view the impact of tourism development on individuals positively. Positive social contact experiences align with residents’ subjective expectations regarding personal utility during contact and communication [48]. This personal utility may include economic benefits like profits, customer cultivation, and word-of-mouth communication, as well as psychological benefits such as a sense of social connection, positive emotions, and recognition as a friendly host. According to Bimonte and Punzo [51], the quality and nature of interactions significantly influence residents’ perceptions of tourism on themselves. Thus, a harmonious relationship resulting from positive social contact reinforces residents’ perception that personal benefits outweigh recognized costs [21].

Residents’ assessment of the impact of tourism development, whether positive or negative, serves as a central variable in predicting resident attitudes and support for tourism. Compared to the more commonly used variable of perceived positive tourism impact in prior studies, personal benefit emerges as a more direct motivational element in real-life interactions, motivating residents to consider citizenship behavior. This is supported by Coghlan’s [52] study, where personal benefits were identified as a key motivator for altruistic volunteer tourism activities. Ribeiro et al. [50] and Woosnam et al. [53] validated the hypothesis that personal economic benefits directly influence residents’ pro-tourism development behaviors. Additionally, Xie, Zhao, and Ma’s [9] study examined the chain process of social interaction, personal benefits, and community citizenship behavior from the perspective of value co-creation. Therefore, the present study proposes the following hypotheses:

**H2a.**  *Resident–tourist interaction behavior can positively influence perceived benefits.*

**H2b.**  *Resident–tourist interaction quality can positively influence perceived benefits.*

**H3.**  *Personal benefits can positively influence community citizenship behavior.*

**H4.**  *Personal benefits play a significant mediator role in the relationship between positive contact and community citizenship behavior.*

#### 2.3.2. Sympathetic Understanding

Empathy stands out as a classic other-oriented motivation in determining altruistic behaviors, as evidenced by prior research [11,54]. Scholars often differentiate between empathy and sympathy, highlighting empathy’s emphasis on transpersonal thinking and identification with the other person, while sympathy involves concern from a third-party perspective [55]. However, Woosnam’s theory of emotional solidarity interprets residents’ sympathetic understanding towards tourists as an understanding of how tourists feel, equating residents’ empathy for tourists with sympathy [56]. The role of empathy in tourism is gaining rapid attention, particularly in comprehending intersubjective relationships in diverse tourism encounters [57].

Woosnam’s emotional solidarity theory provides insights into understanding the link between positive resident–tourist contact and sympathetic understanding. According to the theory, residents’ shared beliefs, behaviors, and interactions with tourists significantly predict their experienced emotional solidarity, fostering sympathetic understanding. Positive contact between the two groups encourages residents to be more empathetic toward tourists, as the latter expresses a desire to understand local culture and preserve local ways of life [56]. Additionally, intergroup contact theory suggests that individuals with qualified social contact perceive each other more positively, yielding better outcomes [37]. Pera et al.’s [58] study on Airbnb customers revealed that direct interaction with the head of the household, in contrast to those using Booking, fosters a concrete, informal, and potentially emotional interaction that helps customers experience a sense of home. This feeling, in turn, stimulates empathy, reducing the likelihood of customers leaving a negative review even after a less-than-ideal experience.

Furthermore, the powerful explanatory role of empathy in prosocial behavior is well documented [11,14,43]. With increased attention to empathy in tourism, various conceptual and empirical studies have explored its role in several prosocial behavior contexts. For instance, Kim and Koo [13], and Yin et al. [33] examined empathetic concern from a motivational lens, affirming its role in determining tourists’ pro-environmental behavior. In Li et al.’s [59] study, tourists’ empathy significantly moderated the effect of tour guides’ service quality on tourists’ pro-tour guide tendencies, supporting empathy as an altruism trigger. Additionally, Li, Liu, and Wei [60] demonstrated a chain process wherein hosts’ sincere interaction encouraged tourists’ sympathetic understanding, predicting tourists’ environmentally responsible behavior. Therefore, according to the above, the following hypotheses were proposed:

**H5a.**  *Resident–tourist interaction behavior can positively influence sympathetic understanding.*

**H5b.**  *Resident–tourist interaction quality can positively influence sympathetic understanding.*

**H6.**  *Sympathetic understanding can positively influence community citizenship behavior.*

**H7.**  *Sympathetic understanding plays a significant mediator role in the relationship between positive contact and community citizenship behavior.*

#### 2.3.3. Place Identity

The present study posited that residents’ identity with the destination functions as an other-oriented motivation influencing CCB. As per Proshansky [61], place identity is a significant concept in tourism, capturing one’s emotional attachment to a place, encompassing ideas, beliefs, preferences, feelings, values, goals, and behavioral tendencies. A robust emotional connection with the place can lead to an altruistic focus on the needs of others [62].

Social interaction plays a pivotal role in shaping place attachment [21]. It is acknowledged that emotional ties to the community result from both individual internal processes and external social processes [63]. When interacting with tourists, residents have the opportunity to reevaluate the local area through the perspective of others. Positive social interactions contribute to a deeper understanding of local culture, the environment, tourism development, etc., which is internalized into personal consciousness and becomes a significant force in the development or reinforcement of place identity. This identity gives rise to a place-oriented motivation, propelling residents to actively engage in behaviors that benefit the tourist destination [45].

Individuals with a higher level of place identity demonstrate a greater willingness to undertake actions that support the place. Beyond predicting residents’ support for tourism development, prior studies offer empirical evidence of the impact of place identity on individuals’ prosocial behaviors. For instance, Wan, Shen, and Choi [44] concluded that place identity plays a crucial positive role in motivating environmentally responsible behavior. Similarly, Lai, et al. [45] suggested that residents’ self-identity can serve as a motivating force for their role as destination ambassadors. Through positive interactions with tourists, residents are more likely to perceive a positive identity level, casting themselves as proud hosts, thus motivating further efforts to reinforce this identity through beneficial behaviors. Consequently, the study proposes the mediating hypothesis that place identity acts as a bridge between positive resident–tourist contact and community citizenship behavior.

**H8a.**  *Resident–tourist interaction behavior can positively influence place identity.*

**H8b.**  *Resident–tourist interaction quality can positively influence place identity.*

**H9.**  *Place identity can positively influence community citizenship behavior.*

**H10.**  *Place identity plays a significant mediator role in the relationship between positive contact and community citizenship behavior.*

**Figure 1:**
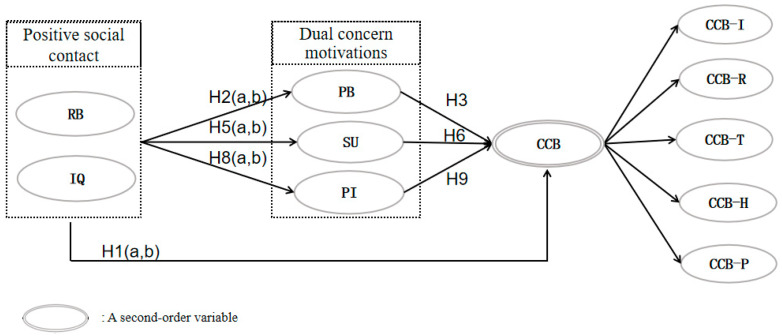
Study conceptual model. Note 1: RB: interaction behavior; IQ: interaction quality; PB: personal benefits; SU: sympathetic understanding; PI: place identity; CCB: community citizenship behavior; CCB-I: involving behavior; CCB-R: recommending behavior; CCB-T: tourist-helping behavior; CCB-H: resident-helping behavior; CCB-P: protecting behavior. Note 2: The mediated hypotheses were not shown in the model.

The conceptual model is presented in Figure 1.

## 3. Study Design

### 3.1. Study Case

The selected research site is Kaifeng, a renowned cultural tourism city in Henan, China. Boasting a history spanning over 4100 years, Kaifeng served as the capital for various Chinese dynasties. It is particularly recognized for its tourist attractions centered around royal culture, folk traditions, delectable snacks, and iconic figures from the Song Dynasty, making it a prime destination for history enthusiasts and global travelers. Kaifeng is a small city with most of its tourist attractions within the old town, distributed around the ancient city wall. Due to limited space, residential areas, and attractions are in close proximity. Additionally, facilitated by social media, tourists now embark on distinctive journeys to explore resident’ preferred, time-honored restaurants, allowing them to immerse themselves in authentic local life. This geographical arrangement, with overlapping living and tourist activities, provides residents, especially those in the vicinity, with substantial exposure to tourists. Consequently, social contact between these two groups is notably more frequent compared to other tourist destinations. The chosen study site is well-suited for examining residents’ perceptions and responses to social interactions with tourists.

Furthermore, the tourism industry in Kaifeng plays a pivotal role in bolstering the city’s economy, generating employment, preserving cultural heritage, and enhancing the overall well-being of the community. Recent statistics reveal that the industry structure ratio between the primary, secondary, and tertiary sectors is 14.3:38.7:47.0, with comprehensive tourism revenue reaching CNY 22.1 billion (Kaifeng Government Statistics in 2022). Given the substantial impact of tourism development in Kaifeng, residents are more inclined to view tourism and related matters positively, driven by collective interests. Therefore, the chosen case site proves to be highly suitable for the study.

### 3.2. Measurement

A self-administered questionnaire was designed following the below steps. Firstly, items of the initial questionnaire were chosen from well-examined scales. Positive social contact was evaluated based on two dimensions: interaction behavior of six items [23,64] and interaction quality of five items [2,65]. Personal benefits from tourism development contained four items adapted from Wang and Pfister [66] and Nunkoo and So [1]. Sympathetic understanding, more relevant to the tourism interaction context, was measured according to Woosnam and Norman’s [56] emotional solidarity concept. Five items were selected as the measures of place identity [45,63]. A total of 26 items scattered in 5 dimensions covered community citizenship behavior, including involving behavior (6 items), recommending behavior (5 items), tourist-helping behavior (5 items), resident-helping behavior (4 items), and protecting behavior (6 items) [5,6]. Then, based on the contextual adjustments made to the initial questionnaire items, the study employed a back-translation method to ensure the semantic accuracy of the item descriptions. All items were evaluated with a 5-point Likert-type scale, with one being “strongly disagree” or “never” and five representing “strongly agree” or “always”. At last, demographic information, including gender, age, and education level, was also collected.

### 3.3. Data Collection and Sample Demographic Characteristic

Before administering the official questionnaire, a pre-survey was conducted by the research team comprising eight trained college students majoring in tourism management from 17 March to 31 March 2023, to confirm the validity of the questionnaire. Utilizing a site-specific random distribution, 70 samples from residents of Kaifeng City were retrieved. Using SPSS data analysis, reliability assessments for the questionnaire scales were conducted, including Cronbach’s alpha, KMO values, and standard factor loading. Specifically, three items were excluded due to low factor loading coefficients and significant covariance issues in the dimension of resident–tourist interaction behavior, while three items were retained. This resulted in a cumulative variance of 75.2%, KMO = 0.765, and Cronbach’s alpha = 0.834. The factor loading coefficients for the remaining latent variables were all above 0.7, and Cronbach’s alpha coefficients were all higher than 0.7, indicating strong validity.

Refinements to the formal questionnaire were made based on the pre-survey data and feedback from respondents (see Table 1). The official research took place from 13 April to 31 May 2023, in residential areas of Kaifeng City (e.g., parks and squares). On the basis of strict adherence to the fundamental norms of research ethics, the survey team initially screened samples by randomly intercepting respondents and verifying their local residency status and experience with tourist interactions. After explaining the survey’s anonymous and academic use, respondents were invited to complete the questionnaire following their approval. To ensure successful completion, minor assistance was provided, including clarifications on questions and small tokens of appreciation. Out of the 400 questionnaires, 366 were selected as valid after excluding those that were partially completed, those that were completed for a brief amount of time, and those that checked the same option on at least eight items, resulting in a validity rate of 91.5%.

Among the 366 valid samples, the demographic characteristics of the respondents are outlined in Table 2. The predominant age group was between 18 and 25 years, with 111 respondents (30.3%), and 26 and 35 years, with 93 respondents (25.4%). The majority of respondents obtained a college or undergraduate degree (198 respondents, 54.1%), followed by those with a high school/associate degree (86 respondents, 23.5%). The proportion of respondents who have resided in Kaifeng for more than 20 years and 1–5 years was approximately equal, accounting for 33.1% of the sample, respectively.

### 3.4. Study Method 

The study primarily used SPSS 26.0 and Smart PLS 4.0 for data analysis. Instead of using Analysis of Moment Structures (AMOS), the study used partial least squares structural equation modeling (PLS-SEM), which is more appropriate for exploratory research and can handle complicated models with smaller sample sizes. Firstly, data screening, demographic characteristics, and sample reliability and validity tests were achieved through descriptive analysis and factor analysis in SPSS. Based on this, the study employed Smart PLS to perform the PLS-SEM algorithm to assess the model’s validity. At last, the hypothesized causal relationships were testified using bootstrapping analyses.

Additionally, Harman’s single-factor test was performed using SPSS. According to the criterion that the eigenvalue of the first factor obtained from the rotation of all items is greater than 1, the explained variance of the first factor is 10.35%, significantly lower than the critical value of 40% [67]. Therefore, it is evident that the sample data do not exhibit a significant common method bias issue.

## 4. Results

### 4.1. Exploratory Factor Analysis

Before using Smart PLS, the study conducted exploratory factor analysis in SPSS to assess the model’s validity. Utilizing the maximum variance approach, the community citizenship behavior scale was further refined by removing four items (RCB1, RCB16, RCB20, and RCB26) with factor loadings significantly below 0.7. Subsequently, with the updated measurement model, the standardized factor loadings of all items ranged between 0.695 and 0.911. The Cronbach’s α for each construct exceeded 0.7, validating the suitability to proceed to the next step (detailed results available in Table 1). 

### 4.2. Measurement Model Analysis

The PLS-SEM algorithm was employed in the study to derive essential values, such as standardized factor loadings, AVE values, CR values, and correlated coefficients, forming the foundation for a comprehensive evaluation of the research model’s validity [68]. Specifically, the repeated indicator approach was utilized for the analysis of community citizenship behavior, a second-order construct. 

As outlined in Table 3, the factor loading for each item was notably higher than 0.7, except for RCB 25, which exhibited a factor loading of 0.683. With the CR values for each latent variable surpassing 0.839 and the AVE ranging from 0.550 to 0.764, meeting the recommended criteria, the measurement model demonstrated favorable convergent validity [69]. Subsequently, Table 4 presents the outcomes of the discriminant validity assessment. Adhering to the Fornell–Lacker criterion, the square root of the AVE for each construct should exceed the correlations with other constructs and the Heterotrait/monotrait ratio method, indicating that the ratio between the two constructs should be below 0.9. These results provided robust evidence of excellent discriminant validity [70].

### 4.3. Structural Model Analysis

To ensure the accuracy of the path analysis results by avoiding collinearity issues, the study examined the collinearity of the model using variance inflation factor (VIF) values before hypothesis testing. The VIF values for all items ranged from 1.293 to 3.764, well below the threshold of 5, indicating the absence of multicollinearity issues [71].

**Figure 2:**
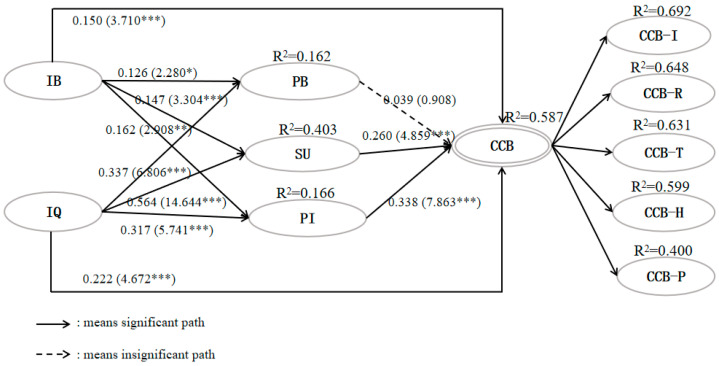
Hypotheses testing results of direct path. Note: *p* < 0.05 *; *p* < 0.01 **; *p* < 0.001 ***.

The hypothesized causal relationships were then tested using the bootstrapping method (bootstrapping = 5000). Figure 2 and Table 5 illustrate that both resident–tourist interaction behavior and quality significantly influenced community citizenship behavior at the *p* < 0.001 level, supporting H1a and H1b. Furthermore, interaction behavior had a significant effect on personal benefits, sympathetic understanding, and place identity at varying levels of significance: β_H2a: RB→PB_ = 0.126 (*p* < 0.05); β_H5a: RB→SU_ = 0.147 (*p* < 0.001); β_H8a: RB→PI_ = 0.162 (*p* < 0.01), suggesting that H2a, H5a, and H8a were supported. Similarly, at the level of *p* < 0.001, the causal relationships between interaction quality and personal benefits (β_H2b: IQ→PB_ = 0.337), sympathetic understanding (β_H5b: IQ→SU_ = 0.564), and place identity (β_H8b: IQ→PI_ = 0.317) were confirmed, supporting H2b, H5b, and H8b. In the path leading to CCB, the significant roles of sympathetic understanding (β_H6: SU→CCB_= 0.260, *t* = 4.859***) and place identity (β_H9: PI→CCB_= 0.338, *t* = 7.863***) were confirmed. Surprisingly, personal benefits did not prove to be a predictor of CCB (β_H3: PB→CCB_ = 0.039, *p* < 0.05). Therefore, H6 and H9 were statistically supported, while H3 was rejected.

The study further examined the significance of indirect effects based on the direct effect results. As shown in Table 5, personal benefits failed to mediate the relationship between positive social contact and community citizenship behavior due to its non-significant influence on community citizenship behavior (*p*_H4a: RB→PB→RCB_ > 0.05, *p*_H4b: IQ→PB→RCB_ > 0.05). Sympathetic understanding (β_H7a: RB→SU→RCB_ = 0.038) and place identity (β_H10a: RB→PI→RCB_ = 0.055) demonstrated their respective mediating roles in the relationship between resident–tourist interaction behavior and community citizenship behavior at *p* < 0.01 level. In contrast, sympathetic understanding (β_H7b: IQ→SU→RCB_ = 0.146) and place identity (β_H10b: IQ→PI→RCB_ = 0.107) played more prominent mediating roles in the relationship between resident–tourist interaction quality and community citizenship behavior, both in terms of the strength of the effect and the level of path significance (*p* < 0.001). Based on these findings, H7 and H10 were verified, while H4 was rejected.

## 5. Conclusions and Discussion

The present study focused on residents’ perceptions of their interactions with tourists, aiming to explore the role of personal benefit from tourism development, sympathetic understanding, and place identity in determining community citizenship behavior within the egoistic–altruistic framework. The empirical data validated the hypotheses, affirming that positive social contact with tourists influences CCB either directly or indirectly by fostering other and place-focused motivations.

Importantly, our study revealed that positive contact with tourists significantly captures residents’ attention at personal, mutual, and place levels, generating corresponding motivations related to CCB. These motivations manifested in perceived personal benefits from tourism development, sympathetic understanding with tourists, and identity with the place. These results align with the existing literature describing how positive contact can yield favorable outcomes from several levels [21,64,72]. Furthermore, sympathetic understanding emerged as the primary motivating factor during positive resident–tourist contact, surpassing personal benefits and place identity. Consistent with Woosnam and Norman’s [56] perspective, positive interactions contribute significantly to residents’ positive attitudes and emotions, fostering empathy, enjoyment, and pride, which, in turn, lead to altruistic behaviors [73]. 

Furthermore, among the three categories of motives influencing CCB—self-oriented, other-oriented, and place-oriented—both sympathetic understanding and place identity emerged as significant predictors, while self-oriented motives (personal benefits) did not receive confirmation. The results suggest that altruistic motives (vs. egoistic motives) hold more sway than egoistic motives in explaining CCB when interacting positively with tourists, supporting Zhang, et al.’s [17] study in predicting residents’ pro-environmental behaviors to some extent. The results of this study also validate the significance of sympathetic understanding and place identity in a range of pro-tourism behaviors, as seen from the perspective of the residents [18,45,60]. Notably, the present study underscores the importance of considering the antecedents of motivation, which dynamically evolve in response to the context [74]. The findings, in contrast to Rodríguez, Pérez, and Alonso [75], confirm the importance of the context in which motivation is generated as noted in Ling and Xu [20]’s study.

Moreover, this study demonstrated that residents’ positive contact with tourists significantly encourages them to participate in CCB, which strongly endorsed Wang, Xiong, and Gage [39] ‘s research. The findings of the mediation effect emphasize residents’ indirect role in fostering CCB by cultivating sympathetic understanding or strengthening place identity during interactions. This logic aligns with research on how resident–tourist interactions can stimulate tourists’ positive behaviors [21,60,64,76].

### 5.1. Theoretical Implication

This study makes significant theoretical contributions to our understanding of resident behaviors at destinations, particularly within the framework of egoistic–altruistic motivations in tourism. Firstly, it expands on the limited research concerning residents’ perceptions of social interactions and various behaviors by empirically investigating how residents’ positive contact with tourists, encompassing interaction behavior and quality, influences community citizenship behavior. Existing studies predominantly approach resident–tourist interactions from the tourists’ perspective, analyzing their impact on the tourists’ experiences, attitudes, and subsequent behaviors [72,76]. However, such interactions profoundly shape residents’ perceptions of tourists, the formation of stereotypes, and attitudes toward tourism development. Consequently, this study responds to the insights of Kim, Duffy, and Moore [22] and Gong, Detchkhajornjaroensri, and Knight [77]. Furthermore, by spotlighting residents’ voluntary and altruistic citizenship behaviors, the research addresses the notion that residents can actively contribute to tourism development [2,3,22], thus broadening the scope of destination residents’ behavioral research. 

The second significant contribution of this research involves investigating the mechanism behind the development of residents’ community citizenship behavior through the lens of egoistic–altruistic motivations. While attitude factors are commonly explored in resident behavior research, the egoistic–altruistic motivation framework provides a natural alignment with altruistic community citizenship behavior, thereby expanding the research scope beyond conventional approaches. Drawing inspiration from the well-established egoistic–altruistic motivation framework in organizational behavior and prosocial behavior studies [11,24,78], this study advocates for applying this framework in tourism by further distinguishing altruistic motivations into other-focused elements (e.g., sympathetic understanding) and place-focused elements (e.g., place identity). Furthermore, the results of the study contribute to the ongoing debate about their relative importance in existing research [11,17,50].

Lastly, this study advances the understanding of egoistic and altruistic motivations in determining citizenship behavior by introducing a precursor to motivation formation—positive contact. Previous research has produced conflicting findings, with some asserting the dominance of altruistic factors over egoistic ones [17], while others highlight the reverse [50]. Our study recognizes that motivations can vary based on external stimuli and individual psychological perceptions, making it challenging to prioritize one motive without specific contextual insights. Consequently, this study aims to reconcile the mixed findings in studies exploring the impact of egoistic and altruistic motivations on encouraging community citizenship behavior at the individual interaction level.

### 5.2. Practical Implication

Community citizenship behavior plays a crucial role as residents proactively engage in tourism development, providing a vital supplement for sustainable growth in destination tourism. To guide and regulate resident citizenship behavior effectively, this study offers practical recommendations for local organizations and tourism managers in three key areas.

Firstly, recognizing the significant impact of positive resident–tourist contact on CCB, it is imperative to enhance the likelihood of such positive interactions. Strategies should focus on boosting interaction behavior and quality among residents and tourists, particularly in cultural destinations. Tourism managers can employ soft measures, including promoting a hospitable host image, fostering a sense of assistance among destination residents, and acknowledging and incentivizing resident engagement with tourists. These initiatives aim to cultivate a positive attitude toward interaction, ultimately elevating the behavior and quality of these engagements. Additionally, advocating for responsible tourism among tourists presents another avenue to achieve positive contact. Managers can utilize official and social channels to disseminate educational materials, such as pamphlets or short videos, educating and guiding tourists to explore destinations with courtesy and respect, fostering meaningful interactions, valuable communication, and an environment of positive interaction and mutual respect. 

Secondly, the study reveals that sympathetic understanding with tourists can evoke CCB, suggesting that enhancing residents’ understanding of tourists may promote pro-tourism behaviors. Destination managers should actively encourage empathy and perspective-taking by facilitating the shared use of recreational spaces between residents and tourists. Simultaneously, efforts should be directed towards the development of a ‘near-psychological distance perception’, centered on elements like ‘shared experiences and moral/cultural identity’, to deepen the emotional connection between the two groups. Managerial initiatives, such as the design of public spaces or communal events like community centers, markets, or festivals, can encourage shared understanding and connection between residents and tourists, enabling residents to better comprehend the tourist experience.

Finally, in line with previous research findings, place identity emerges as a crucial antecedent variable influencing community citizenship behavior. Destination managers can implement various measures to promote residents’ identity with the destination. For instance, the development of cultural activities and traditions that reflect cultural soft power can nurture residents’ deeper connection and commitment to the destination. When residents take pride in their culture, they are more inclined to contribute wholeheartedly to the positive development of local tourism. Additionally, destination managers should provide appropriate support to residents without excessively catering to tourists, addressing residents’ emotional needs for safety and comfort in their local living environment. This approach is vital for strengthening residents’ sense of place.

### 5.3. Limitations

Interestingly, our initial empirical findings indicated the lack of significance in personal benefit from tourism development when predicting CCB, deviating from expectations. This study posits that this unexpected result may stem from the limited scope of research scenarios in previous studies, and it suggests that residents’ citizenship behaviors might be more intricately tied to altruistic considerations within positive resident–tourist interaction contexts. However, this assertion remains speculative, necessitating further research to validate its accuracy. Additionally, future investigations should broaden their scope to encompass a diverse range of scenarios, exploring specific contexts influencing residents’ motivation for citizenship behaviors, including online social environments [79] and the impact of citizenship behaviors of others [22,80], to deepen the understanding of the interplay between altruism and egoism in tourism research.

Furthermore, the three motivational variables (personal benefit, sympathetic understanding, and place identity) utilized in this study are grounded in individual-level perceptions among residents. However, the impact of tourism development on a destination is complex and multifaceted, leading to nuanced attitudes towards tourism and related elements. Residents, as an informally organized group, sometimes experience the prevailing influence of group dynamics superseding individual factors in specific scenarios. Consequently, future research could integrate motivational factors at the collective level, such as community social capital and tolerance, to collectively explore residents’ citizenship behavior [15,74].

Lastly, this study relied on a self-reported survey for analysis, potentially resulting in an overestimation of respondents’ assessments of their engagement in CCB due to social desirability bias. Therefore, future studies should consider incorporating supplementary sources of information, such as in-depth interviews, to provide a more comprehensive understanding of residents’ psychological perceptions.

## Figures and Tables

**Table 1:** The results of exploratory factor analysis.

Construct and Items	Standard Factor Loading	Mean	Standard Deviation	Cronbach α	KMO
Resident–tourist interaction behavior (RB)				0.798	0.765
RB4 Offering help to tourists when necessary	0.714	4.243	0.708		
RB5 Being politely to tourists	0.911	4.303	0.647		
RB6 Showing courtesy to tourists	0.903	4.358	0.636		
Resident–tourist interaction quality (IQ)				0.831	0.838
IQ1 Tourists treat me as a friend	0.781	2.863	1.023		
IQ2 My interactions with tourists are positive and useful	0.704	3.691	0.786		
IQ3 I enjoy interacting with tourists	0.852	3.328	0.939		
IQ4 Tourists enjoy interacting with me	0.827	3.219	0.909		
IQ5 Tourists share their hometown culture with me	0.697	3.536	1.042		
Personal benefits from tourism development (PB)				0.796	0.747
PB1 Tourism development has provided jobs for me or my family	0.770	3.934	0.891		
PB2 Tourism development has led to many festivals or events	0.855	3.760	0.916		
PB3 Tourism development has provided more leisure opportunities	0.800	4.139	0.810		
PB4 Tourism development has enhanced community services	0.725	3.631	0.982		
Sympathetic understanding (SU)				0.799	0.782
SU1 I identify with visitors in Kaifeng	0.852	3.309	0.878		
SU2 I have a lot in common with Kaifeng’s tourists	0.786	3.273	0.894		
SU3 I feel affection towards visitors in Kaifeng	0.767	3.536	0.854		
SU4 I understand visitors in Kaifeng	0.753	3.836	0.862		
Place identity (PI)				0.922	0.888
PI1 I strongly identify with my community	0.849	3.874	0.947		
PI2 Living and working in my community means a lot about who I am	0.831	3.937	1.018		
PI3 I feel my community is a part of me	0.906	3.787	1.045		
PI4 I feel attached to my community	0.887	3.923	0.964		
PI5 I feel a sense of belonging to my community	0.894	3.751	1.089		
Community citizenship behavior (CCB)					
Involving behavior (CCB-I)				0.870	0.818
RCB2 I actively participate in various tourism-related training programs and development meetings	0.852	3.139	1.045		
RCB3 I actively participate in voluntary activities to promote tourists’ travel experience	0.848	3.232	1.031		
RCB4 I actively participate in cultural protection and promotion in Kaifeng (e.g., history culture, heritage)	0.776	3.593	0.984		
RCB5 I report tourism development related problems initiatively	0.807	3.268	1.042		
RCB6 I make suggestions to Kaifeng on tourism development when necessary	0.771	3.612	1.017		
Recommending behavior (CCB-R)				0.866	0.777
RCB7 I say positive things about Kaifeng to others	0.722	4.109	0.806		
RCB8 I actively promote the image of Kaifeng outside	0.824	4.128	0.825		
RCB9 I promote the tourism features and products of Kaifeng to others	0.797	4.096	0.852		
RCB10 I encourage my relatives and friends to visit Kaifeng	0.848	3.937	1.029		
RCB11 I actively recommend others to visit Kaifeng	0.844	3.945	1.007		
Tourist-helping behavior (CCB-T)				0.746	0.732
RCB12 Whenever I encounter visitors, I willingly help them with directions and so on	0.780	3.962	0.727		
RCB13 Whenever I encounter visitors, I try my best to help them	0.742	4.172	0.709		
RCB14 I am always helpful towards tourists	0.725	3.527	0.940		
RCB15 Whenever I encounter visitors, I try to be friendly to them	0.764	4.019	0.715		
Resident-helping behavior (CCB-H)				0.713	0.763
RCB17 I lend a hand to other residents in need	0.820	3.497	1.026		
RCB18 I share information and resources with other residents	0.751	3.361	0.998		
RCB19 I avoid creating problems for other residents	0.819	3.366	1.075		
Protecting behavior (CCB-P)				0.795	0.801
RCB21 I keep the city environment clean	0.730	4.298	0.641		
RCB22 I protect the city’s tourism resources	0.795	4.290	0.692		
RCB23 I protect the city’s image	0.764	4.374	0.685		
RCB24 I actively protect the heritages in Kaifeng (e.g., ancient architecture, and folk culture)	0.721	4.380	0.706		
RCB25 I strictly obey the city’s rules and regulations related to tourism development	0.695	4.497	0.704		

**Table 2:** The demographic characteristics of the study.

Demographics	Frequency	Percentage %	Demographics	Frequency	Percentage %
Gender			Length of stay		
Male	151	41.3	1–5 years	121	33.1
Female	215	58.7	6–9 years	54	14.8
Age			10–14 years	39	10.7
18–25 years	111	30.3	15–19 years	31	8.5
26–35 years	93	25.4	More than 20 years	121	33.1
36–45 years	76	20.8	Monthly income (RMB)		
46–55 years	50	13.7	Less than 3000	140	38.3
56–65 years	26	7.1	3001–6000	138	37.7
66 years and above	10	2.7	6001–10,000	59	16.1
Educational background			10,001–15,000	21	5.7
Junior School or below	46	12.6	15,001–30,000	7	1.9
High school/Associate	86	23.5	More than 30,000	1	0.3
College/Undergraduate	198	54.1			
Master or above	36	9.8	N = 366		

**Table 3:** The results of measurement model: reliability and validity.

Construct and Items	Standardized Factor Loading	AVE	CR	Construct and Items	Standardized Factor Loading	AVE	CR
Resident–tourist interaction behavior (RB)				Community citizenship behavior (CCB)			
RB4	0.770	0.715	0.882	Involving behavior (CCB-I)			
RB5	0.881			RCB2	0.849	0.658	0.906
RB6	0.881			RCB3	0.845		
Resident–tourist interaction quality (IQ)				RCB4	0.777		
IQ1	0.782	0.600	0.882	RCB5	0.806		
IQ2	0.701			RCB6	0.776		
IQ3	0.853			Recommending behavior (CCB-R)			
IQ4	0.824			RCB7	0.728	0.654	0.904
IQ5	0.701			RCB8	0.826		
Personal benefits from tourism development (PB)				RCB9	0.795		
PB1	0.714	0.618	0.866	RCB10	0.846		
PB2	0.855			RCB11	0.842		
PB3	0.788			Tourist-helping behavior (CCB-T)			
PB4	0.782			RCB12	0.795	0.567	0.840
Sympathetic understanding (SU)				RCB13	0.737		
SU1	0.853	0.624	0.869	RCB14	0.729		
SU2	0.765			RCB15	0.749		
SU3	0.784			Resident-helping behavior (CCB-H)			
SU4	0.754			RCB17	0.825	0.636	0.839
Place identity (PI)				RCB18	0.740		
PI1	0.850	0.764	0.942	RCB19	0.823		
PI2	0.832			Protecting behavior (CCB-P)			
PI3	0.905			RCB21	0.718	0.550	0.859
PI4	0.889			RCB22	0.791		
PI5	0.892			RCB23	0.777		
				RCB24	0.734		
				RCB25	0.683		

**Table 4:** The results of discriminant validity assessment.

**Fornell–Larcker Criterion**						
**Construct**	**PB**	**PI**	**SU**	**IQ**	**RB**	**RCB**
PB	0.786					
PI	0.292	0.874				
SU	0.458	0.421	0.790			
IQ	0.385	0.379	0.620	0.775		
RB	0.255	0.283	0.363	0.382	0.846	
CCB	0.380	0.585	0.612	0.583	0.435	0.597
**Heterotrait/Monotrait Ratio (HTMT)**						
**Construct**	**PB**	**PI**	**SU**	**IQ**	**RB**	**RCB**
PB						
PI	0.332					
SU	0.573	0.483				
IQ	0.459	0.427	0.757			
RB	0.329	0.328	0.445	0.472		
CCB	0.428	0.634	0.711	0.661	0.512	

**Table 5:** Hypotheses testing results of indirect path. Note: *p* < 0.05 *; *p* < 0.01 **; *p* < 0.001 ***.

Hypotheses	β	*t*-Value	*p* Values	95%CI	Results
H1a: RB → CCB	0.150	3.710	***	(0.069, 0.229)	Supported
H1b: IQ → CCB	0.222	4.672	***	(0.129, 0.315)	Supported
H2a: RB → PB	0.126	2.280	*	(0.018, 0.236)	Supported
H2b: IQ → PB	0.337	6.806	***	(0.239, 0.436)	Supported
H3: PB → CCB	0.039	0.908	0.364	(−0.043, 0.124)	Rejected
H5a: RB → SU	0.147	3.304	***	(0.060, 0.236)	Supported
H5b: IQ → SU	0.564	14.644	***	(0.490, 0.640)	Supported
H6: SU → CCB	0.260	4.859	***	(0.153, 0.363)	Supported
H8a: RB → PI	0.162	2.908	**	(0.053, 0.269)	Supported
H8b: IQ → PI	0.317	5.741	***	(0.210, 0.422)	Supported
H9: PI → CCB	0.338	7.863	***	(0.255, 0.422)	Supported
H4a: RB → PB → RCB	0.005	0.757	0.449	(−0.005, 0.021)	Rejected
H7a: RB → SU → RCB	0.038	2.686	**	(0.014, 0.069)	Supported
H10a: RB → PI → RCB	0.055	2.800	**	(0.018, 0.095)	Supported
H4b: IQ → PB → RCB	0.013	0.903	0.367	(−0.016, 0.043)	Rejected
H7b: IQ → SU → RCB	0.146	4.522	***	(0.085, 0.212)	Supported
H10b: IQ → PI → RCB	0.107	4.390	***	(0.064, 0.159)	Supported

## Data Availability

The raw data supporting the conclusions of this article will be made available by the authors on request. The data are not publicly available due to the data also forming part of an ongoing study and cannot be publicly shared for the time being.
